# Ischemic Postconditioning Fails to Protect against Neonatal Cerebral Stroke

**DOI:** 10.1371/journal.pone.0049695

**Published:** 2012-12-12

**Authors:** Pierre-Louis Leger, Philippe Bonnin, Thao Nguyen, Sylvain Renolleau, Olivier Baud, Christiane Charriaut-Marlangue

**Affiliations:** 1 Univ Paris Diderot, Sorbonne Paris Cité, INSERM, U676, Paris, France; 2 Univ Paris Diderot, Sorbonne Paris Cité, AP-HP, Hôpital Lariboisière, Physiologie Clinique, Explorations-Fonctionnelles, Paris, France; 3 Univ Paris Diderot, Sorbonne Paris Cité, INSERM, U965, Paris, France; 4 UPMC-Paris6, AP-HP, Hôpital Armand Trousseau, Service de Réanimation pédiatrique, Paris, France; 5 Univ Paris Diderot, Sorbonne Paris Cité, AP-HP, Service de Réanimation néonatale et pédiatrique, Hôpital Robert Debré, Paris, France; University of Queensland, Australia

## Abstract

The lack of efficient neuroprotective strategies for neonatal stroke could be ascribed to pathogenic ischemic processes differentiating adults and neonates. We explored this hypothesis using a rat model of neonatal ischemia induced by permanent occlusion of the left distal middle cerebral artery combined with 50 min of occlusion of both common carotid arteries (CCA). Postconditioning was performed by repetitive brief release and occlusion (30 s, 1 and/or 5 min) of CCA after 50 min of CCA occlusion. Alternative reperfusion was generated by controlled release of the bilateral CCA occlusion. Blood-flow velocities in the left internal carotid artery were measured using color-coded pulsed Doppler ultrasound imaging. Cortical perfusion was measured using laser Doppler. Cerebrovascular vasoreactivity was evaluated after inhalation with the hypercapnic gas or inhaled nitric oxide (NO). Whatever the type of serial mechanical interruptions of blood flow at reperfusion, postconditioning did not reduce infarct volume after 72 hours. A gradual perfusion was found during early re-flow both in the left internal carotid artery and in the cortical penumbra. The absence of acute hyperemia during early CCA re-flow, and the lack of NO-dependent vasoreactivity in P7 rat brain could in part explain the inefficiency of ischemic postconditioning after ischemia-reperfusion.

## Introduction

Perinatal arterial ischemic stroke is a major cause of later neurological disabilities including cerebral palsy, epilepsy, and cognitive deficiencies [1]. Tissue plasminogen activator (tPA) is the only approved agent capable of improving reperfusion following ischemia in the adult brain [2]. However, no safe molecule improving reperfusion is currently available to protect the immature brain. Until now, only molecules targeting apoptotic cell death, such as caspase inhibitors [3], [4] and PARP-1, an enzyme facilitating DNA relaxation and repair [5], have demonstrated reduced infarct volume in a well-established neonatal P7 rat stroke model [6], that mimics the main features of ischemic damage in third trimester-human fetus [7].

In the adult rat brain, restoration of blood flow to the ischemic territory is characterized by a significant hyperemia within the penumbra occurring immediately after occlusion release. This is followed by a post-ischemic hypoperfusion, described as the “no-reflow phenomenon” [8]. Ischemic postconditioning (postC), defined as serial mechanical interruptions of blood flow at reperfusion, was recently demonstrated to be a harmless procedure attenuating cerebral blood flow (CBF) disturbances in adult. Ischemic postC either interrupts hyperemia [9] or shortens hyperperfusion time [10] and reduces infarct volume. These studies suggest that abrupt reperfusion may exacerbate ischemic injury [11], and that ischemic postC was a valuable strategy to reduce infarct.

We first investigated whether ischemic postC might reduce infarct volume in the ischemic P7 rat brain. As we found striking differences compared to adult, we then sought to characterize reperfusion during the early re-flow. We analyzed the spatiotemporal profile of CBF changes using both 2D-color-coded ultrasound imaging and laser Doppler flowmetry. To further understand these differences, we thus examined nitric oxide (NO)-mediated cerebrovascular reactivity under CO_2_ and/or inhaled NO exposure.

## Methods

### Ethics Statement

All experiments complied with ethical guidelines of Robert Debré Hospital Research Council Review Board (A75-19-01), INSERM and the ARRIVE guidelines (http://www.nc3rs.org/ARRIVE), approving this study.

### Neonatal ischemia-reperfusion

Ischemia was induced in Wistar P7 rat pups (17–21 g; Janvier, Le Genest St-Isle, France) as previously described [6] and adapted to gas anesthesia [12]. Briefly, thermoregulated (37.0±0.5 C°) and anesthetized pups [under 1% isoflurane in O_2_/N_2_O (1∶3)] were exposed to left middle cerebral artery electocoagulation (MCAo) combined with a transient (50 min) and concomitant occlusion of both common carotid arteries (CCA). After recovery, pups were transferred to their mothers. Rat pups were sacrificed at 72 hours.

### Ischemic postconditioning

**Figure 1:**
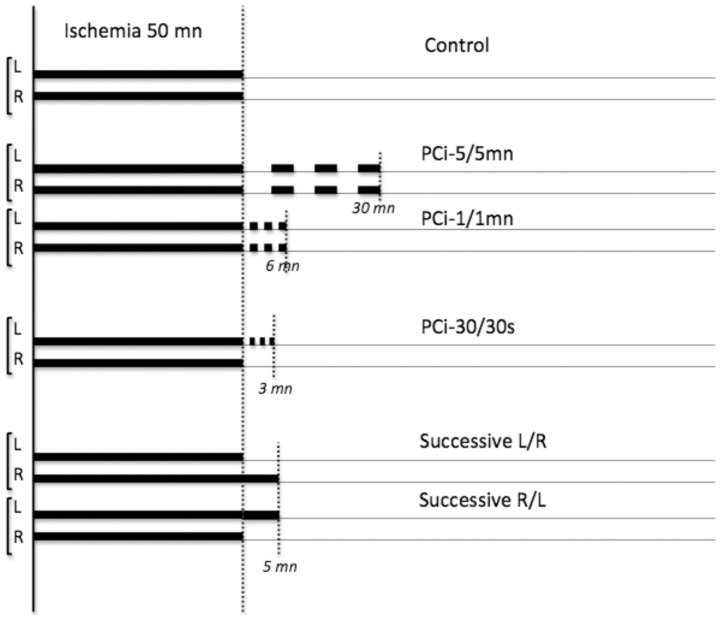
Protocols for cerebral ischemia without and with postconditioning. Animals were divided into three groups. All animals were subjected to MCA electrocoagulation, and bilateral CCA were transiently (50 min) occluded 2 min later. Re-flow was initiated by CCA occlusion release in controls (n = 17 in the first set, n = 11 in the second set and n = 11 in the third set of experiments). Postconditioning with 3 cycles (occlusion/reperfusion) of 30 s (n = 11), 1 (n = 18) or 5 (n = 12) min was performed on both or only left CCA within 15 s after the initial CCA reperfusion. Alternative reperfusion was initiated by first occlusion release of the left (L/R, n = 11) or of the right (R/L, n = 12) CCA followed by occlusion release of the other CCA.

Animals under anaesthesia were randomly assigned to ischemic postC procedure or control groups (Fig. 1). After the 50 min bilateral CCA occlusion, postC was induced by re-occluding either bilateral CCA or only left CCA. The re-occlusion times were 30 sec, 1 min to 5 min. CCA(s) were released for the same duration. The procedure was repeated for 3 cycles. In another set of experiments, alternative reperfusion was generated by controlled release of first the right or left CCA 5 min before the release of the other one. Two investigators, who were blind to the treatment group, determined the size of the lesion in each animal (n = 111).

### Ultrasound imaging

Thermoregulated rat pups (n = 10) were subjected to ultrasound measurements under isoflurane [0.5% in O_2_/N_2_O (1∶3)] anesthesia using an echograph (Vivid 7, GE Medical Systems ultrasound®, Horten, Norway) equipped with a 12-MHz linear transducer [12]. Time-average mean blood-flow velocities (mBFV) were measured in the left intracranial internal carotid artery (ICA) and in the basilar trunk (BT) before surgery, during ischemia (at 40 min, n = 111 to exclude animals with very high mBFVs [12]) and monitored from 1 to 15 min after removal of the CCA occlusion (n = 10). Heart rates were measured and reflected changes in cardiac output, as ventricular stroke volume is quite invariable in newborns. According to the values of mBFVs found in the BT during ischemia [12], animals were included or not in the postconditioning experiments. In another sets of experiments, mBFVs (in the left ICA) were measured during reperfusion in animals subjected to unaltered reperfusion (n = 5), 1/1 min postC (n = 6), and/or to alternative reperfusion (n = 6).

Cerebral vasoreactivity was evaluated, by measuring mBFVs in the BT and both ICAs, in rat pups by exposure to 5% CO_2_ in the inhaled N_2_/O_2_ gas mixture through a facemask, or exposure to exogenous inhaled NO [(iNO) using the iNOvent system (Ikaria, Clinton, NJ)] at 20-ppm through a facemask (n = 5–6 each condition in the two experiments).

### Cortical regional CBF (rCBF)

In six animals (thermoregulated and anaesthetized) placed in the prone position the left calvarium was exposed by incision and left cortical rCBF measurements were made by laser Doppler flowmetry (Moor Instruments Ltd, Axminster, UK) by using the stainless MP7b probe (Moor Instruments Ltd) [12]. Doppler probe was placed on the skull (∼2 mm posterior and ∼3 mm lateral to the bregma). Relative changes in rCBF in the penumbra were recorded in 3 regions of interest over a period of 5 min in basal, after MCAo, during ischemia, and during the first 20 min after reperfusion and averaged; rCBF measurements were normalized to baseline in each animal.

### Physiological parameters

Blood was collected by intracardiac puncture and gases (pH, pO_2_, pCO_2_) were measured in basal and after reperfusion, and after inhalation of gases (5% CO_2_ or iNO) by the means of a blood gas analyzer (Ciba-Corning 248). Continuous transcutaneous pCO_2_ was measured by the means of a pO_2_-pC0_2_ monitor (Philips Medical System, IntelliVue MP40 neonatal, Boeblingen, Germany) with pO_2_/pCO_2_ sensors (Radiometer Medical ApS, Bronshoj, Denmark).

### Measurement of infarct volume

Infarct volumes were determined on 14 cresyl violet-stained coronal sections (at 500 µm interval) using an image analyzer (Image-Pro, Paris, France). Cortical infarct volumes on the left hemisphere were measured at 72 hours after ischemic onset, and expressed as a percentage of the total ipsilateral cortical and sub-cortical volume (at this time point there is no residual edema [6]), as the lesion is mainly present in the cortex and white matter [12].

### Statistical analysis

Values are expressed as mean±SD. ANOVA for repeated measurements and *post hoc* paired Student's *t*-test were used to analyze differences in mean BFV at different time points. Infarct volumes were analyzed using ANOVA and the *post hoc* Newman-Keuls test for multiple comparaisons. Changes in physiological parameters were analyzed using paired Student's *t*-test.

## Results

One hundred eleven animals underwent the ischemic procedure. Twelve animals were excluded as “no-lesion”, distributed in all groups. All of them exhibited a very high increase in mean blood-flow velocity (mBFV) in the basilar trunk (≥200%) during ischemia according to our previous report [12].

### Postconditioning did not reduce infarct size

**Figure 2:**
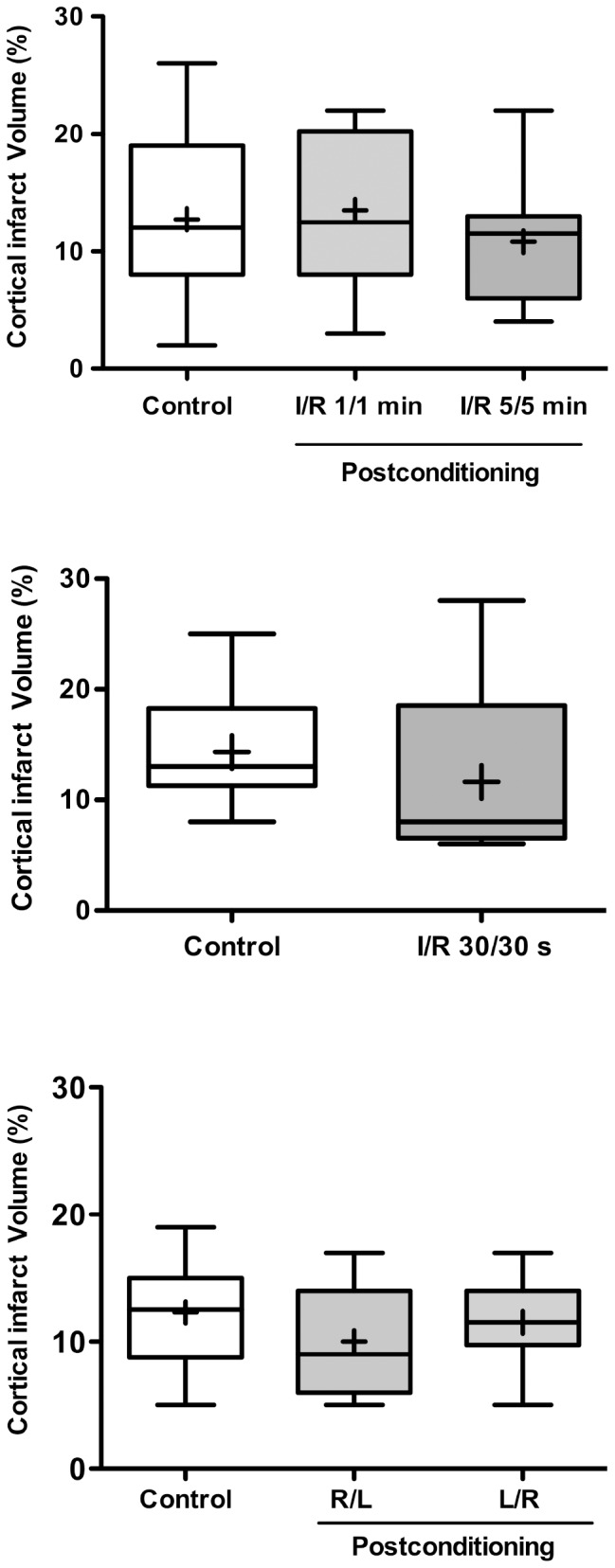
Postconditioning did not reduce infarct size. Infarct size was measured 3 days after ischemia. **A**: Infarct size measured in animals subjected to postconditioning with 3 cycles of occlusion/reperfusion of 1 and 5 min on both CCA. **B**: Infarct size measured in animals subjected to postconditioning with 3 cycles of occlusion/reperfusion of 30 s on the left CCA. **C**: Infarct size measured in animals subjected to alternative reperfusion. The median (horizontal bar) and the mean (cross) were indicated. No significant difference was detected in the different groups.

In control animals subjected to left MCA and transient bilateral CCA occlusion mean infarct size measured was 12.8±7.2% (median = 12%) at 72 hours (n = 15, 2 animals died) (Fig. 2A). The size of the lesion did not differ after postC combining 3 cycles of occlusion/re-flow on both CCA of 1/1 min (mean = 13.5±6.3%, median = 12%, n = 16, 2 animals died) or 5/5 min (mean = 10.8±5.1%, median = 12.5%, n = 12, 2 animals died). As the lesion in our model is only observed on the left hemisphere, we also evaluated the impact of postC 30 s/30 s on the left CCA only. No reduction in the infarct size was obtained in postC animals (mean = 11.6±9.2%, %, n = 11) (Fig. 2B), compared to the infarct size in their respective control animals (mean = 14.3±6.4%, median = 13%, n = 11). In the last set of experiments (Fig. 2C), alternative reperfusion by the release of occlusion first in the right CCA followed by left CCAo release produced a mean lesion volume of 10.0±4.3% (R/L, median = 9%, n = 11), whereas first release in the left CCA followed by the right CCAo release produced a mean lesion volume of 11.5±3.3% (L/R, median = 11.5%, n = 12), both not significantly different from the respective control group (mean = 12.3±4.3%, median = 12.5%, n = 11).

### Absence of hyperemia during early reperfusion

**Figure 3:**
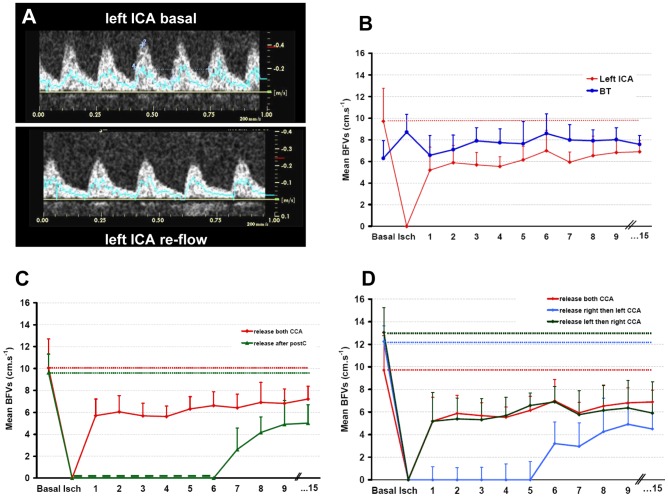
Changes in arterial cerebral blood flow in animals subjected to neonatal ischemia. **A**: spectral analyses of blood-flow velocity waveforms acquired with pulsed Doppler in the left ICA before ischemia (basal, left) and at 3 min after re-flow (right). **B**: Time course of mBFV in the left ICA and in the basilar trunk (BT) during reperfusion. Mean BFVs were plotted before ischemia, during ischemia (at 40 min) and during the first 15 min of the reperfusion. Note that mBFVs in the left ICA at the reperfusion did not reach the level of its mean value in basal condition. In the BT, mBFVs were increased during ischemia (*p<0.05 *vs* basal) and returned to basal values at the reperfusion. **C–D**: Time course of mBFV in the left ICA during reperfusion in animals subjected to neonatal ischemia and/or postconditioning. **C**: Mean BFVs were plotted before ischemia (basal), during ischemia (at 40 min) and during the first 15 min of the reperfusion after release on both CCA (ischemic controls, red line, n = 5), and after 1/1 min postC (3 cycles, green line, n = 6). **D**: Mean BFVs were plotted before ischemia (basal), during ischemia (at 40 min) and during the first 15 min of the reperfusion after release on both CCA (ischemic controls, red line, n = 6), after release occlusion on left CCA then right CCA 5 min later (green line, n = 5), and after release on the right then left CCA 5 min later (blue line, n = 5). Data are expressed as mean±SD.

To explain the absence of protection conferred by ischemic postconditioning, and based on our hypothesis we then investigated the temporal profile of early re-flow. In control pups (n = 10) during ischemia and whereas the two CCA are occluded, mBFV increased to 138.4±51.4% in the basilar trunk explaining animal-model variability [12]. During early reperfusion, mBFV in the BT slightly decreased to 127.2±31.7% (NS *vs* basal values, Fig. 3B) and remained stable. Re-flow in the left ICA occurred immediately after clip removing (Fig. 3A–B). Mean BFV in the left ICA reached 63±40% of basal values after 1 min of re-flow (p<0.01 vs basal), gradually recovering to 72±21% (p<0.01 vs basal) at 15 min. Animals exhibited stable heart rates before (352±42 bpm), during ischemia (322±30 bpm) and 15 min after re-flow (370±24 bpm).

After the 1/1 min postC protocol (3 cycles of occlusion/reperfusion), mBFVs in the left ICA at recovery were blunted and gradually increased to the level of unaltered reperfusion in ischemic animals without postC at 15 min after postC (Fig. 3C). Release left then right and/or release right then left CCA (alternative reperfusion) did not also modify mBFV time-course with similar values at 5 and/or 10, and 15 min of re-flow in the left ICA (Fig. 3D).

**Figure 4:**
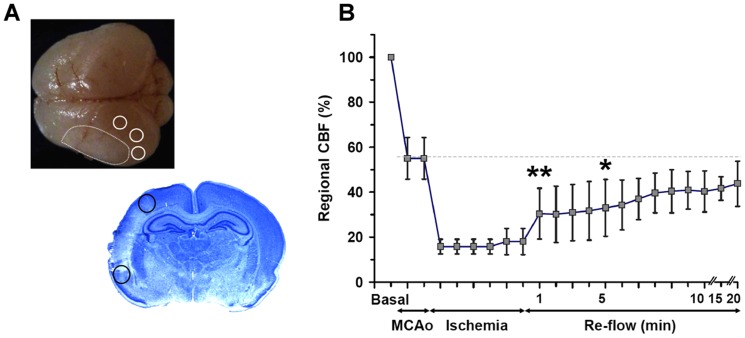
Laser Doppler monitoring during reperfusion. **A**: Representative brain from a pup killed at 48 hours after ischemia showing a pale delineated lesion (white dotted line) and 3 regions of interest (ROI), and representative cresyl violet-stained section showing a cortical infarct and 2 ROI for rCBF measurements in the penumbra with the laser probe. **B**: Changes in rCBF (mean of 3 ROI ± S.D) in 6 animals subjected to ischemia-reperfusion. Upon MCA occlusion, the rCBF dropped to 55±8% of baseline, and additional bilateral CCA occlusion further decreased rCBF to 18±5%. After CCA release, a gradual reperfusion was observed from 30±11% at 1 min to 44±9% 20 min after. *p<0.05, ** p<0.01 *vs* basal.

After MCA occlusion, rCBF measured by laser Doppler declined to 55±8%, and further declined to 18±5% of baseline after bilateral CCA occlusion (Fig. 4, n = 6). After bilateral CCA occlusion release, rCBF gradually increased from 30±11% at 1 min to 44±9% at 20 min (Fig. 4B) and recovered to 55±10% in a few hours after re-flow (not shown).

### Cerebrovascular reactivity in the P7 rat

Inhalation with the 5% hypercapnic gas mixture induced an increase in pCO_2_ (from 56.4±7.5 in basal to 77.5±6.9 mmHg, p<0.01) and transcutaneous pCO_2_ (from 55.7±7.2 in basal to 73.2±6.7 mmHg, p<0.0001, see Fig. S1), and a markedly acidosis (pH from 7.342±0.047 in basal to 7.137±0.003, p<0.01) in P7 rat pups in basal conditions. Values in pO_2_ remained unchanged (from 33.3±5.9 in basal to 33.7±8.3 mmHg, NS), and suggested a venous contamination of our blood samples. Hypercapnia induced by 5% CO_2_ did not produce an increase in mBFVs in the 3 pre-Willis arteries either in basal P7 or after 15 min of reperfusion in injured P7 rat pups (n = 6 each condition, Table 1).

**Table 1:** Reactivity in the pre-Willis cerebral arteries as assessed by ultrasound imaging in naive rat pups (n = 5) and 15 min after ischemia-reperfusion (n = 5) during normoxia (air), hypercapnia (5% CO2), and return to normoxia.

		Air	5% CO_2_	5% CO_2_
**Naive**	BT	7.4±1.2	6.2±2.3	7.3±1.0
	Left ICA	10.6±1.5	8.3±2.7	10.2±1.5
	Right ICA	11.7±2.0	8.2±4.0	10.4±0.7
	Heart rates	319±34	230±21	205±24
**Reperfusion**	BT	9.0±0.7	7.8±0.9	8.8±0.6
	Left ICA	6.6±3.0	5.1±1.7	6.4±2.5
	Right ICA	3.5±0.7	2.8±0.6	3.4±0.6
	Heart rates	292±41	261±7	250±10

Data represent mean BFVs measured in cm.s−1 in the three pre-Willis cerebral arteries (±SD): BT, basilar trunk; ICA, internal carotid artery. Heart rates are given in bpm.

Inhalation with 20-ppm NO (iNO, n = 6), a dose that we reported to be delivered to the brain, and to be efficient during ischemia by increasing collateral recruitment and reducing infarct volume [13] did not change pH and pCO_2_ found in basal (see above), but significantly increased pO_2_ (from 33.3±5.9 in basal to 41.5±7.3 mmHg, p<0.01). Inhaled NO induced a similar gradual time-course of mBFVs in the left ICA to that obtained without iNO exposure with similar mBFVs during reperfusion (Table S1).

## Discussion

This is the first evidence demonstrating why ischemic postconditioning could not mediate neuroprotection in a neonatal ischemia-reperfusion model. A progressive and incomplete reperfusion (no hyperemia) occurred in the cortex during the early re-flow, and an absence of NO-mediated cerebrovascular reactivity found in P7 rat pups could be ascribed to processes differentiating adults and neonates. Although much of our knowledge on the pathophysiology of cerebral hypoxia-ischemia and ischemia is based on extensive experimental studies in adult animal models, major differences occur in the immature brain, including energy metabolism and nutrient transport, glutamate excitotoxicity, oxidative stress and apoptosis, and impact on responses to, and recovery to hypoxia and/or ischemia [14], [15].

Monitoring mBFV by ultrasound imaging indicates no hyperemia over basal values in the pre-Willis large arteries during reperfusion. In the basilar trunk, mBFVs increased during ischemia then returned to basal values at re-flow. In the left ICA, early re-flow was effective but did not reach maximal values and did not exhibited an hyperemia episode as detected in adult stroke [8]. We recently reported [12] that the rise in mBFVs in the basilar trunk, whereas the two CCA were occluded (ischemia), reflects 1) the efficiency of collateral support through the circle of Willis as described in rodents [16] and humans [17]. Interestingly, whatever the range of mBFV rise in the basilar trunk (responsible for the variability of the lesion size), a progressive but incomplete reperfusion without hyperemia was observed in the left internal carotid artery in all animals, strongly suggesting that collateral recruitment still remains efficient at early re-flow, as no thrombus was observed in isolated carotid arteries after occlusion release (not shown). Primary collateral pathway through the circle of Willis could provide an immediate diversion of cerebral blood flow towards ischemic regions, although the secondary pathway through the cortical anastomoses (extend between vascular networks from anterior and middle, and, posterior and middle cerebral arteries), even when anatomically present, requires time to develop. Magnitude of blood-flow redistribution through the primary collateral pathway depends of the relative changes in local vascular resistances and blood pressure gradients between the different vascular territories supplied by the different cerebral arteries branched to the circle of Willis. Thus, blood-flow redistribution in the primary collateral network also depends from the secondary cortical collateral pathway. We recently reported that neuronal NO synthase (nNOS) had a greater role than endothelial NOS (eNOS) in collateral recruitment during ischemia in the P7 rat [18]. In addition, uncoupling of perivascular NOS, probably eNOS due to acquired relative tetrahydrobiopterin deficiency occurred after neonatal hypoxia-ischemia in P7 rat pups [19]. Therefore, the absence of hyperemia at re-flow could result from the absence and/or reduction of an efficient endothelial function in the P7 rat brain. Inhaled NO exposure during reperfusion, increased mBFVs to a level similar to that obtained in the left ICA without iNO, highly suggesting that the magnitude of vasodilation was achieved, most likely mediated by the neuronal nNOS during the ischemic period [18]. In contrast, opening of the cortical collaterals in adult rats depends on several compensatory hemodynamic, metabolic, and neural mechanisms [20]. This important difference between immature and mature hemodynamic responses may be consistent with data demonstrating that neonatal animals had larger caliber midline collaterals than adult animals after bilateral CCA occlusion [21].

Monitoring cerebral perfusion in the penumbra by laser Dopppler indicates that reperfusion is gradual during the first 20 min of re-flow and reaches the level obtained with permanent MCA occlusion in most of animals after several hours. Studies in adult rats have demonstrated an episode of transient hyperemia at the early stage of reperfusion after a focal or global ischemia [22]. Gradual CBF recovery avoid hyperemia and reduced the infarct volume by 30% in adult rats after controlled CCAs release (first ipsilateral CCA and 2 min later contralateral CCA) [22]. This hyperemia could be related to the great vasodilation of the local vascular network because collateral recruitment in adult was only effective after a few hours [23], [24]. In contrast, we did not find evidence of such hyperemia at early re-flow in our rat pups, in agreement with data showing that the CBF responses to hypoxia-ischemia and reperfusion differ depending on postnatal age, with hyperemia in juvenile but not neonatal rats [25]. Probably this is a reflection of a smaller vasodilation magnitude in pups consecutive to the very early (within 10 min) effective collateral recruitment during ischemia [12] as compared to adults, and its maintenance during early re-flow. This smaller vasodilation magnitude could also be explained by both paCO_2_ and transcutaneous pCO_2_ values found in P7 rat pups, higher than those measured in adult rats [26], suggesting that vasodilation could not be efficient in this pCO_2_ and acidosis range. Specific vascular responses to ischemia between neonatal and adult rat brain could be also explained by differences in hypoxia tolerance and energy metabolism [27]. In piglets (1–2 weeks of life), hyperperfusion after ischemia and reperfusion was observed to the brain stem and caudate nucleus [28], suggesting a species-dependent cerebrovascular reactivity. However, cerebral oxygen consumption at 10 min of reperfusion was unchanged in piglets but reduced in pigs [28]. Finally, it was reported that newborn cerebrovascular responses are largely NO independent, and NO becomes more important with maturation [29].

In conclusion, our data indicate that ischemic postconditioning is not a valuable strategy to reduce infarct size in the neonatal brain, at least in part, due to the absence of hyperemia at early re-flow. As the reperfusion is diminished in neonates, we cannot totally exclude that the difference in CBF level between the ischemic and reperfusion phase was too blunted, which may account in the ineffectiveness of postC whatever postconditioning protocols (as evaluated in the adult rat). However, the absence of vasodilation in the left ICA induced by CO_2_ or inhaled NO, may in part exclude to test other ischemic postconditioning paradigms.

## Supporting Information

Figure S1 **Transcutaneous pCO_2_ in a vigil rat.** Continuous transcutaneous pCO_2_ was measured by the means of a PO_2_-PC0_2_ monitor (Philips Medical System, IntelliVue MP40 neonatal, Boeblingen, Germany) in vigil P7 rat pups, after isoflurane (1%) anesthesia, after 5% CO_2_ under anesthesia, and after return to normoxia (stop of CO_2_ and isoflurane) (n = 5, each condition). Note that PCO_2_ increased from 55.7±7.2 (basal) to 73.2±6.7 mm Hg under 5% CO_2_, and returned to basal values (57.7±4.3 mm Hg) after the stop of CO_2_ and isoflurane.
(TIF)

Table S1 **Mean blood-flow velocities in the left ICA (cm.s^−1^) during ischemia and reperfusion.** Inhaled NO (given at the beginning of the reperfusion) induced milar mBFVs in the left ICA to that obtained without iNO exposure.
(DOC)

## References

[1] Golomb MR, Garg BP, Saha C, Azzouz F, Williams LS (2008) Cerebral palsy after perinatal arterial ischemic stroke. J Child Neurol 23: 279–286. 18305317 10.1177/0883073807309246

[2] Fisher M, Bastan B (2008) Treating acute ischemic stroke. Curr Opin Drug Discov Devel 11: 626–632. 18729014

[3] Renolleau S, Fau S, Goyenvalle C, Joly LM, Chauvier D, et al (2007) Specific caspase inhibitor Q-VD-OPh prevents neonatal stroke in P7 rat: a role for gender. J Neurochem 100: 1062–1071. 17166174 10.1111/j.1471-4159.2006.04269.x

[4] Chauvier D, Renolleau S, Holifanjaniaina S, Ankri S, Bezault M, et al (2011) Targeting neonatal ischemic brain injury with a pentapeptide-based irreversible caspase inhibitor. Cell Death Dis 2: e203. 21881605 10.1038/cddis.2011.87 PMC3186905

[5] Ducrocq S, Benjelloun N, Plotkine M, Ben-Ari Y, Charriaut-Marlangue C (2000) Poly(ADP-ribose) synthase inhibition reduces ischemic injury and inflammation in neonatal rat brain. J Neurochem 74: 2504–2511. 10820212 10.1046/j.1471-4159.2000.0742504.x

[6] Renolleau S, Aggoun-Zouaoui D, Ben-Ari Y, Charriaut-Marlangue C (1998) A model of transient unilateral focal ischemia with reperfusion in the P7 neonatal rat: morphological changes indicative of apoptosis. Stroke 29: 1454–1460 discussion 1461. 9660403 10.1161/01.str.29.7.1454

[7] Northington FJ (2006) Brief update on animal models of hypoxic-ischemic encephalopathy and neonatal stroke. Ilar J 47: 32–38. 16391429 10.1093/ilar.47.1.32

[8] Sutherland BA, Papadakis M, Chen RL, Buchan AM (2011) Cerebral blood flow alteration in neuroprotection following cerebral ischaemia. J Physiol 589: 4105–4114. 21708904 10.1113/jphysiol.2011.209601 PMC3180571

[9] Zhao H, Sapolsky RM, Steinberg GK (2006) Interrupting reperfusion as a stroke therapy: ischemic postconditioning reduces infarct size after focal ischemia in rats. J Cereb Blood Flow Metab 26: 1114–1121. 16736038 10.1038/sj.jcbfm.9600348

[10] Wang JY, Shen J, Gao Q, Ye ZG, Yang SY, et al (2008) Ischemic postconditioning protects against global cerebral ischemia/reperfusion-induced injury in rats. Stroke 39: 983–990. 18239163 10.1161/STROKEAHA.107.499079

[11] Aronowski J, Strong R, Grotta JC (1997) Reperfusion injury: demonstration of brain damage produced by reperfusion after transient focal ischemia in rats. J Cereb Blood Flow Metab 17: 1048–1056. 9346429 10.1097/00004647-199710000-00006

[12] Bonnin P, Leger PL, Deroide N, Fau S, Baud O, et al (2011) Impact of intracranial blood-flow redistribution on stroke size during ischemia-reperfusion in 7-day-old rats. J Neurosci Methods 198: 103–109. 21420433 10.1016/j.jneumeth.2011.02.030

[13] Charriaut-Marlangue C, Bonnin P, Gharib A, Leger PL, Villapol S, et al (2012) Inhaled Nitric Oxide Reduces Brain Damage by Collateral Recruitment in a Neonatal Stroke Model. Stroke 10.1161/STROKEAHA.112.664243 22949477

[14] Renolleau S, Fau S, Charriaut-Marlangue C (2008) Gender-related differences in apoptotic pathways after neonatal cerebral ischemia. Neuroscientist 14: 46–52. 17971506 10.1177/1073858407308889

[15] Vannucci SJ, Hagberg H (2004) Hypoxia-ischemia in the immature brain. J Exp Biol 207: 3149–3154. 15299036 10.1242/jeb.01064

[16] Longa EZ, Weinstein PR, Carlson S, Cummins R (1989) Reversible middle cerebral artery occlusion without craniectomy in rats. Stroke 20: 84–91. 2643202 10.1161/01.str.20.1.84

[17] van Laar PJ, Hendrikse J, Klijn CJ, Kappelle LJ, van Osch MJ, et al (2007) Symptomatic carotid artery occlusion: flow territories of major brain-feeding arteries. Radiology 242: 526–534. 17255422 10.1148/radiol.2422060179

[18] Bonnin P, Leger PL, Villapol S, Deroide N, Gressens P, et al (2012) Dual action of NO synthases on blood flow and infarct volume consecutive to neonatal focal cerebral ischemia. Exp Neurol 236: 50–57. 22531298 10.1016/j.expneurol.2012.04.001

[19] Fabian RH, Perez-Polo JR, Kent TA (2008) Perivascular nitric oxide and superoxide in neonatal cerebral hypoxia-ischemia. Am J Physiol Heart Circ Physiol 295: H1809–1814. 18676689 10.1152/ajpheart.00301.2007 PMC2593505

[20] Liebeskind DS (2003) Collateral circulation. Stroke 34: 2279–2284. 12881609 10.1161/01.STR.0000086465.41263.06

[21] Choy M, Ganesan V, Thomas DL, Thornton JS, Proctor E, et al (2006) The chronic vascular and haemodynamic response after permanent bilateral common carotid occlusion in newborn and adult rats. J Cereb Blood Flow Metab 26: 1066–1075. 16395291 10.1038/sj.jcbfm.9600259

[22] Gao X, Ren C, Zhao H (2008) Protective effects of ischemic postconditioning compared with gradual reperfusion or preconditioning. J Neurosci Res 86: 2505–2511. 18438944 10.1002/jnr.21703

[23] Menzies SA, Hoff JT, Betz AL (1992) Middle cerebral artery occlusion in rats: a neurological and pathological evaluation of a reproducible model. Neurosurgery 31: 100–106 discussion 106–107. 1641086 10.1227/00006123-199207000-00014

[24] Liebeskind DS (2009) Stroke: the currency of collateral circulation in acute ischemic stroke. Nat Rev Neurol 5: 645–646. 19953114 10.1038/nrneurol.2009.193

[25] Qiao M, Latta P, Foniok T, Buist R, Meng S, et al (2004) Cerebral blood flow response to a hypoxic-ischemic insult differs in neonatal and juvenile rats. Magma 17: 117–124. 15538659 10.1007/s10334-004-0058-4

[26] Bonvento G, Seylaz J, Lacombe P (1994) Widespread attenuation of the cerebrovascular reactivity to hypercapnia following inhibition of nitric oxide synthase in the conscious rat. J Cereb Blood Flow Metab 14: 699–703. 7520450 10.1038/jcbfm.1994.90

[27] Vannucci RC, Yager JY, Vannucci SJ (1994) Cerebral glucose and energy utilization during the evolution of hypoxic-ischemic brain damage in the immature rat. J Cereb Blood Flow Metab 14: 279–288. 8113323 10.1038/jcbfm.1994.35

[28] Kirsch JR, Helfaer MA, Blizzard K, Toung TJ, Traystman RJ (1990) Age-related cerebrovascular response to global ischemia in pigs. Am J Physiol 259: H1551–1558. 2240252 10.1152/ajpheart.1990.259.5.H1551

[29] Willis AP, Leffler CW (2001) Endothelial NO and prostanoid involvement in newborn and juvenile pig pial arteriolar vasomotor responses. Am J Physiol Heart Circ Physiol 281: H2366–2377. 11709401 10.1152/ajpheart.2001.281.6.H2366

