# Supplementary figures and images for: Ischemic Postconditioning Fails to Protect against Neonatal Cerebral Stroke
Source: PLoS One. 2012 Dec 12;7(12):e49695. doi: 10.1371/journal.pone.0049695 (PMC3520965; doi:10.1371/journal.pone.0049695)

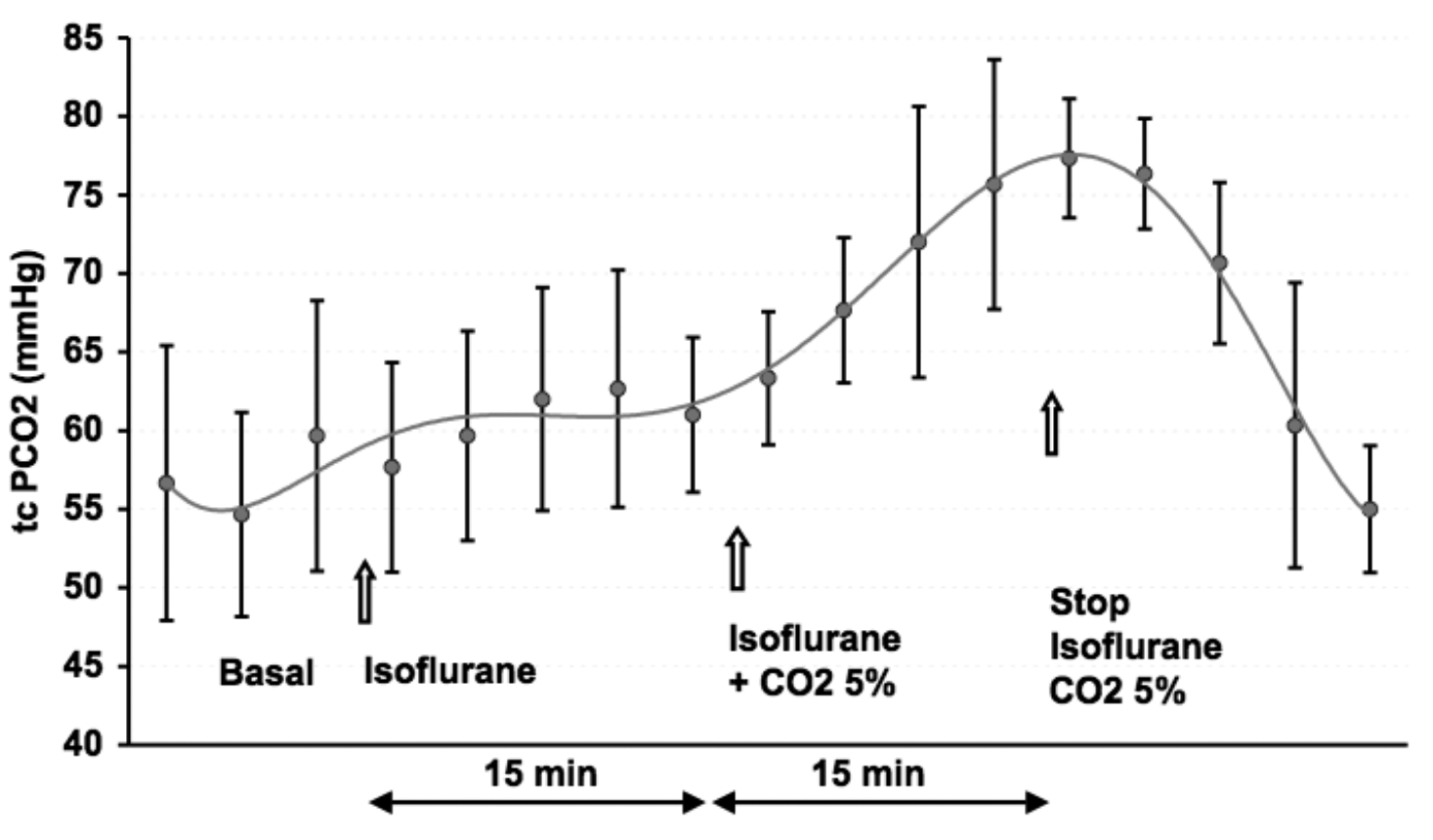

Supplement: Figure S1 — Transcutaneous pCO2 in a vigil rat. Continuous transcutaneous pCO2 was measured by the means of a PO2-PC02 monitor (Philips Medical System, IntelliVue MP40 neonatal, Boeblingen, Germany) in vigil P7 rat pups, after isoflurane (1%) anesthesia, after 5% CO2 under anesthesia, and after return to normoxia (stop of CO2 and isoflurane) (n = 5, each condition). Note that PCO2 increased from 55.7±7.2 (basal) to 73.2±6.7 mm Hg under 5% CO2, and returned to basal values (57.7±4.3 mm Hg) after the stop of CO2 and isoflurane. (TIF) [file pone.0049695.s001.tif]
