# Supplementary material for: Ischemic Postconditioning Fails to Protect against Neonatal Cerebral Stroke
Source: PLoS One. 2012 Dec 12;7(12):e49695. doi: 10.1371/journal.pone.0049695 (PMC3520965; doi:10.1371/journal.pone.0049695)
Supplement: Table S1 — Mean blood-flow velocities in the left ICA (cm.s−1) during ischemia and reperfusion. Inhaled NO (given at the beginning of the reperfusion) induced milar mBFVs in the left ICA to that obtained without iNO exposure. (DOC) [file pone.0049695.s002.doc]

**Table S1**: Mean blood-flow velocities in the left ICA (cm.s^-1^) during ischemia and reperfusion.

|  | **Basal** | **Ischemia** | **Reperfusion** | | | |
| --- | --- | --- | --- | --- | --- | --- |
|  |  |  | 1 min | 5 min | 10 min | 15 min |
| Air | 9.7±3.1 | 0 | 5.2±2.1 | 6.2±1.2 | 6.2±2.2 | 6.9±1.1 |
| Air + iNO 20-ppm | 12.0±2.3 | 0 | 5.3±0.7 | 5.9±1.5 | 6.4±1.6 | 6.6±1.4 |

Inhaled NO (given at the beginning of the reperfusion) induced milar mBFVs in the left ICA to that obtained without iNO exposure.
